# On the Application of Calcium Phosphate Micro- and Nanoparticles as Food Additive

**DOI:** 10.3390/nano12224075

**Published:** 2022-11-19

**Authors:** Joachim Enax, Frederic Meyer, Erik Schulze zur Wiesche, Matthias Epple

**Affiliations:** 1 Dr. Kurt Wolff GmbH & Co. KG, Research Department, Johanneswerkstr. 34-36, 33611 Bielefeld, Germany; 2 Inorganic Chemistry and Center for Nanointegration Duisburg-Essen (CeNIDE), University of Duisburg-Essen, Universitaetsstr. 5-7, 45117 Essen, Germany

**Keywords:** calcium phosphate, nanoparticles, microparticles, food additives

## Abstract

The human body needs calcium and phosphate as essential nutrients to grow bones and teeth, but they are also necessary for many other biochemical purposes (e.g., the biosynthesis of phospholipids, adenosine triphosphate, ATP, or DNA). The use of solid calcium phosphate in particle form as a food additive is reviewed and discussed in terms of bioavailability and its safety after ingestion. The fact that all calcium phosphates, such as hydroxyapatite and tricalcium phosphate, are soluble in the acidic environment of the stomach, regardless of the particle size or phase, means that they are present as dissolved ions after passing through the stomach. These dissolved ions cannot be distinguished from a mixture of calcium and phosphate ions that were ingested separately, e.g., from cheese or milk together with soft drinks or meat. Milk, including human breast milk, is a natural source of calcium and phosphate in which calcium phosphate is present as nanoscopic clusters (nanoparticles) inside casein (protein) micelles. It is concluded that calcium phosphates are generally safe as food additives, also in baby formula.

## 1. Introduction

Calcium phosphate is a common mineral in geology. Furthermore, it is the inorganic mineral in mammalian bone and teeth, also in humans. In bone and dentin, calcium phosphate occurs as nanocrystals in combination with the protein collagen. In tooth enamel, calcium phosphate is the main component (approx. 97 wt% hydroxyapatite) and occurs as micrometer-sized rods [1,2,3,4,5]. Thus, it is often used as a biomaterial in contact with hard tissue [6,7,8], e.g., to coat implants such as endoprostheses or dental implants [9,10,11,12,13] and as a synthetic bone substitution material [14,15,16,17]. In these applications, it is considered to be safe both in a nanoscopic and microscopic dimension [14,18,19,20], because it enhances bone growth and is finally degraded to harmless products, i.e., calcium and phosphate ions, by osteoclasts [21,22,23] or macrophages [24]. In dentistry, calcium phosphate finds application as an active ingredient in toothpaste to restore demineralized enamel and to prevent caries [25,26]. Formulations that contain casein phosphoprotein together with amorphous calcium phosphate are also on the market as “biomimetic” ingredients of toothpaste [26,27,28,29,30]. Calcium phosphate dissolves upon swallowing in the acidic environment of the stomach by dissociation into the constituent calcium and phosphate ions.

Here, we discuss the application of calcium phosphate in food technology, including baby formula, with a special emphasis on (nano)particle-related aspects.

## 2. Calcium Phosphates

There are a number of chemical compounds that fall under the category of calcium phosphates. Chemically, these are all calcium salts of orthophosphoric acid, H_3_PO_4_. Depending on the degree of protonation, the anions of orthophosphoric acid are orthophosphate PO_4_^3−^, monohydrogen phosphate HPO_4_^2−^, and dihydrogen phosphate H_2_PO_4_^−^. Together with the bivalent cation Ca^2+^ and water molecules, a range of different compounds is formed [3,4]. Chemically, salts are ionic solids that are held together by electrostatic interactions (Coulomb forces) between the constituting ions, i.e., cations and anions. Unlike in molecules, there are no covalent bonds between cations and anions; therefore, molecular structures that can sometimes be found for calcium phosphates (e.g., [31]) are not correct. The most important calcium phosphates in biology and food technology are monocalcium phosphate Ca(H_2_PO_4_)_2_ (MCP), dicalcium phosphate CaHPO_4_ (DCP), tricalcium phosphate Ca_3_(PO_4_)_2_ (TCP), and pentacalcium phosphate (better known as hydroxyapatite; HAP), Ca_5_(PO_4_)_3_OH. It is noteworthy that in the literature and on websites, the type of calcium phosphate is often not clearly specified, and that tricalcium phosphate and hydroxyapatite are often confused in statements that are addressed to consumers or the public (e.g., in blogs or manufacturers’ websites).

All calcium phosphates are soluble in acids, typically below a pH of 4 to 5. At a neutral pH, the solubility of hydrogen phosphates (HPO_4_^2−^) and dihydrogen phosphates (H_2_PO_4_^−^) is generally higher than that of orthophosphates (PO_4_^3−^) [4]. At increasing temperatures, the solubility of calcium phosphates increases [3]. The solubility of nanoparticulate calcium phosphate is higher than that of microscopic calcium phosphate due to the higher specific surface area [32]. Upon dissolution in water, the ions that are present in the salt separate due to hydration by the solvent and are present as solvated cations and anions. The solubility is controlled by thermodynamics and usually expressed as the solubility product of the constituting ions [4]. For instance, the solubility product of hydroxyapatite, Ca_5_(PO_4_)_3_OH, is defined as

*c*(Ca^2+^)^5^ · *c*(PO_4_^3−^)^3^ · *c*(OH^−^) = 10^−58.4^ mol^9^ L^−9^ (1)

This holds for pure water as a solvent. In biological media, the ions may form complexes with biomolecules that enhance the solubility of the salt. At low pH, *c*(OH^-^) is decreased, and therefore the solubility increases. It must be stressed that *c*(PO_4_^3−^) is not identical with the total phosphate concentration in a given system but is lower due to protonation to HPO_4_^2−^ and H_2_PO_4_^−^. The fraction of these protonated ions increases at a lower pH and thus increases the solubility at a decreasing pH as well. These are the reasons that all calcium phosphates are soluble in acids, regardless of their chemical formula [4].

As the doses given for phosphates in the literature are expressed in different forms, we give the stoichimetric factors to convert the doses for convenience in the following: *w*(P):*w*(P_2_O_5_):*w*(PO_4_^3−^):*w*(CaHPO_4_):*w*(Ca_3_(PO_4_)_2_):*w*(Ca_5_(PO_4_)_3_OH) = 31:142/2:95:136:310/2:502/3 = 1:2.29:3.06:4.39:5:5.40.

## 3. Application of Calcium Phosphate as Food Additive

The fact that calcium phosphate has been routinely used as a food additive for decades means that recent scientific publications on this topic are rare. We performed a comprehensive literature survey searching for “calcium phosphate”/”hydroxyapatite” and “food”/”beverages”/”nutrient” and selected the appropriate references after inspection of their suitability. With such a broad context, many references did not deal with the topic of this review and were therefore not considered. Additional information is available in the patent literature and in manufacturers’ websites and blogs. As the latter sources can hardly be considered persistent, they are not cited here. However, we have included information from these sources into this review as well. Selected general references on the application of calcium phosphate as a component of toothpastes and dental care products were included as calcium phosphate is also used in these applications (as in food).

Calcium phosphate is mainly used as an additive in flour with up to several wt% (monocalcium phosphate, dicalcium phosphate, tricalcium phosphate) [33]. Here, it serves as a dispersant to help in the baking process and as an acid-neutralizing agent. It also improves the consistency of flour. Crystalline anhydrous calcium phosphate does not change with temperature, i.e., there is no decomposition up to 300-400 °C, depending on the calcium phosphate phase [4]. In particular, microcrystalline tricalcium phosphate (TCP) and hydroxyapatite (HAP) will not change up to 600 °C or more. However, if calcium phosphate is present either as hydrate or in nanoparticulate or amorphous form, it will change upon heating (above about 100 °C) by releasing crystal water or by (re-)crystallizing into a more stable calcium phosphate phase [34,35]. Due to its poor solubility in neutral water, it reacts slowly with water vapor or moisture during storage. Thus, it is well-suited as a food additive. It is tasteless, odorless, and not very hygroscopic. It can neutralize acids without releasing a gas as calcium carbonate, CaCO_3_, or sodium carbonate (soda), as Na_2_CO_3_, would do (i.e., release carbon dioxide, CO_2_, during neutralization). Calcium phosphate is also used as a white pigment to enhance the white cloudiness of soymilk and some nutritional drinks, as well as in fish preparation (see refs. [33,36,37] for a full overview of calcium phosphates as food additives).

After digestion, calcium phosphate provides the ions calcium and phosphate, which are both important in the body, also for the maintenance of strong bones [38,39,40]. The diet is a major source of phosphate for bone formation and also as a substrate for adenosine triphosphate (ATP) synthesis [40]. Both calcium ions and phosphate ions are safe if ingested in reasonable quantities (see below). It has been argued that the administration of calcium phosphate is better suited to preventing osteoporosis than the administration of calcium salts only, because it does not lead to phosphate depletion [39].

In summary, the major effects of calcium phosphate as a food additive are:

- to improve the consistency and processability of flour;
- as an acid regulator;
- as a white pigment; and
- as a nutritional source of calcium and phosphate.

**Figure 1:**
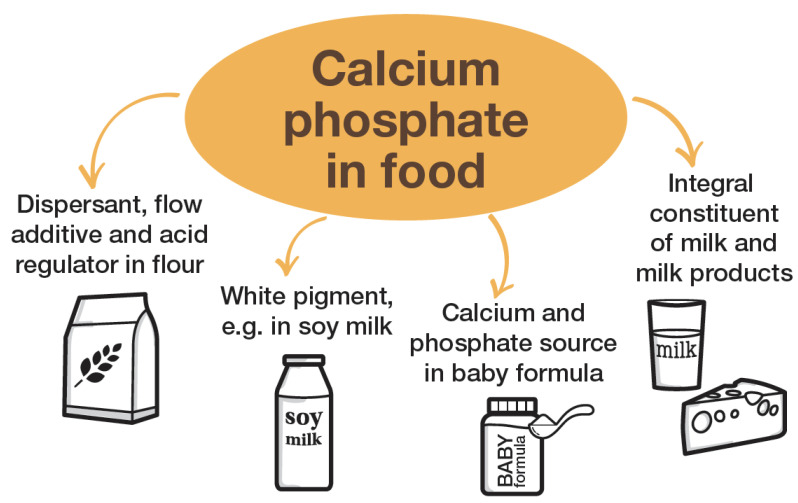
Major applications and occurrence of solid calcium phosphate in food technology.

Figure 1 illustrates the different applications of calcium phosphate in food technology.

## 4. Calcium Phosphate Nanoparticles in Milk

Solid calcium phosphate occurs in milk. Human breast milk contains considerable amounts of calcium and phosphate to supply the infant during breastfeeding: on average, 252 mg L^−1^ calcium and 143 mg L^−1^ phosphorus, with considerable individual variations [41]. Other mammalian milk, e.g., from cows, goats, or sheep, is similar [42]. Notably, the calcium content in human milk is lower than in the milk of other mammals, e.g., cow milk [43]. This is ascribed to the comparatively slow development of human infants in comparison to most animals, requiring a lower calcium delivery rate for skeletal formation [44]. Today, milk and dairy products are the major sources of calcium and phosphorus in human nutrition [38]. In terms of the solubility product of hydroxyapatite, milk is supersaturated with respect to the precipitation of calcium phosphate, but no precipitation occurs due to the complexation of calcium ions by citrate (forming soluble calcium-citrate complexes) and the presence of proteins, which also bind calcium [42,44,45]. The stabilization of calcium phosphate in the form of solid clusters also contributes to the apparent supersaturation of milk with respect to calcium phosphate precipitation. The separation of different calcium species in milk is possible by centrifugation and ultrafiltration [43]. 

A major part of calcium and phosphate in milk is present as very small clusters (nanoparticles) of calcium phosphate, which are embedded in larger particles of the milk protein casein, the so-called casein micelles [42]. These are actually not micelles but protein particles with a typical diameter of 100 nm to 200 nm [43,44,45,46,47]. Casein micelles in cow, buffalo, goat, and sheep milk have a similar size [48]. Each casein micelle contains tens of thousands of casein molecules [49] and thousands of calcium phosphate clusters [45] with a distance of about 18.6 nm apart from each other, as shown by small-angle X-ray scattering (SAXS) [50].

Casein is one of the major proteins in milk (the other main group are whey proteins [51]). It belongs to a family of secreted calcium- (phosphate-) binding phosphoproteins, which also regulate biomineralization, e.g., in bone [52]. Osteopontin (OPN), which is well-known in bone biomineralization, is another member of this protein family [53]. As caseins do not crystallize, their exact structure—even without the calcium phosphate cluster—is unknown and only accessible by alternative methods, e.g., NMR spectroscopy [51]. The isoelectric point of human caseins is 4.3, i.e., agglomeration and coagulation are strongly enhanced below this pH [47].

Incorporated into casein micelles, the calcium phosphate nanoparticles remain solubilized and are available for resorption by the baby consuming the milk. Notably, these particles are extremely small and on the border between clusters and particles. Phosphate groups from the phosphoprotein casein cover their surface and serve as links between the inorganic particle and the organic protein matrix [51,52]. The nanoparticles have a diameter of about 2.5 nm [42] and a molar calcium-to-phosphate ratio of about 2:1 for the inorganic core and about 1:1 if phosphate groups from casein are taken into account as well [45]. Not only are they stabilized by casein themselves, but they are also essential for the integrity and structural stability of the casein micelles in a synergetic way [42,44,45]. 

Chemically, these calcium phosphate nanoparticles can be considered as a solid phase (a salt) and are held together by ionic forces, as in calcium phosphate itself. Such very small calcium phosphate clusters are too small to show X-ray diffraction peaks [54], i.e., they are X-ray amorphous and consist of amorphous calcium phosphate (ACP) [45]. It has been shown that in biological environments, ACP precipitates first [49], followed by crystallization to dicalcium phosphate, octacalcium phosphate, or hydroxyapatite [52,55]. Extended X-ray absorption fine structure (EXAFS) showed a structural similarity to dicalcium phosphate dihydrate, CaHPO_4_·2 H_2_O [54]. By magic-angle spinning (MAS) NMR spectroscopy (^31^P), it was demonstrated that 81% of the organic phosphate and 97% of the inorganic phosphate of a casein micelle in native bovine milk at its original pH of 6.8 occur in an immobile, i.e., quasi-solid state, inside the casein micelles. 

At a decreasing pH (4.8), the calcium phosphate nanoparticles are dissolved and not fully restored after return to the original pH of 6.8 [56]. This points to an uncontrolled re-precipitation inside or outside the casein micelle. In a similar study by ^31^P-MAS-NMR spectroscopy, the effect of the casein proteins to stabilize the calcium phosphate clusters in their amorphous state was shown, including the destabilization of the mineral–protein interface at increasing temperatures [57]. Unfortunately, there is no cryo-electron microscopic structure of casein micelles with incorporated calcium phosphate clusters available. Attempts have been reported to prepare artificial casein micelles by precipitation of calcium phosphate in the presence of casein and citrate [58] and phosphopeptides [59]. In the presence of casein phosphopeptides (see [60] for a general review on this class of compounds and its biological function), calcium phosphate nanoparticles with a diameter of about 2.2 to 2.6 nm are formed [59]. Synthetic assemblies of calcium phosphate and casein are on the market in toothpaste for biomimetic tooth regeneration [27,61,62,63].

The equilibria of calcium and phosphate species in milk have been described by Holt et al. with a model that takes into account all relevant complexation and solubility equilibria [45]. Most calcium in milk is present in dissolved form, mainly with citrate as an anion that acts as a complexing agent [64]. The concentration of solid calcium phosphate (i.e., the nanoparticles) in human milk was estimated to be 0.4 mM calcium, i.e., about 16 mg L^−1^ calcium (about 40 mg L^−1^ calcium phosphate as hydroxyapatite) or about 7% of the total calcium amount in milk [43]. If we tentatively assume a diameter of each calcium phosphate particle of 2.5 nm and the density of hydroxyapatite (3160 kg m^−3^), we compute a volume of each particle of (1.25·10^−9^ m)^3^·4/3·π = 8.2·10^−27^ m^3^ and a weight of 2.6·10^−23^ kg. Thus, at a mass concentration of 40 mg L^−1^ = 4·10^−5^ kg L^−1^, the particle concentration in milk is about 1.5·10^18^ L^−1^, i.e., an astonishingly high number of calcium phosphate nanoparticles.

## 5. Calcium Phosphate Nanoparticles in Blood, Gut, and Saliva

In blood, calcium phosphate occurs in so-called calciprotein particles, which consist of a calcium phosphate core and a protein shell, usually as an intimate mixture of these two components. Typically, they have a size of 100 to 200 nm [65,66,67,68]. Calciprotein particles are stabilized by proteins such as fetuin in blood [69,70]. Similar particles have been discovered in the intestine at high concentrations, probably due to reprecipitation in the neutral/alkaline environment after dissolution in the stomach [71]. In the mouth, calcium phosphate can precipitate from saliva that is supersaturated with respect to calcium phosphate. This may lead to tooth remineralization and also to dental calculus [27]. Hidaka et al. have demonstrated that in the presence of nutrients, amorphous calcium phosphate (ACP) particles are initially formed that later crystallize to hydroxyapatite. The crystallization was enhanced by carbohydrates and oil and constrained by proteins [72]. It has been argued that such calcium phosphate clusters in saliva, blood, and milk are very similar in nature and size, because they are all induced and protected by phosphopeptides and phosphoproteins [52].

In a remarkable article, Hannig and Hannig discussed the occurrence of calcium phosphate nanoparticles that are continuously formed by mechanical wear from tooth enamel during chewing. These are dispersed in the saliva and may help in enamel remineralization and biofilm reduction. Notably, their occurrence was probably higher in historic and prehistoric times, when the abrasion of the tooth by the food, e.g., stone remnants from grain milling, was more prevalent than today [73]. In an interesting experiment, Wang et al. have precipitated calcium phosphate nanoparticles in the presence of different fruit juices, thereby creating a food additive. Although there is no difference to synthetically prepared calcium phosphate particles as a food additive (besides the presence of some organic material from fruits), this shows that the precipitation of calcium phosphate in the presence of fruit juices leads to nanoparticulate solids [74].

## 6. Regulatory Aspects of Calcium Phosphate as Food Additive

Calcium phosphate is generally recognized as safe (GRAS) by the Food and Drug Administration (FDA) [75]. The World Health Organization (WHO) has approved the use of calcium phosphate as a food additive, e.g., as an acidity regulator, a flour treatment agent, a nutrient supplement, and a texturizer and thickener [76]. The advantage of calcium phosphate in comparison to other salts is the fact that it contains both beneficial ions in one compound [77]. Calcium phosphate is approved as a food additive in the EU with the number E341. Interestingly, it is mentioned in E341 only as mono-, di-, and tricalcium phosphate; hydroxyapatite as the most common calcium phosphate is not mentioned in E341, which may explain the occasional confusion in the literature [78]. Tricalcium phosphate cannot be precipitated from water [4], and it is likely that many calcium phosphates used in food technology under the label E341 actually consist of precipitated hydroxyapatite instead of tricalcium phosphate. Incidentally, calcium phosphate phases can only be identified and distinguished by X-ray powder diffraction [79].

Phosphates as food additives, including baby formula and food for medical purposes, were considered in-depth by the EFSA Panel of Food Additives and Flavourings (FAF). The panel concluded that phosphate salts are safe as food additives (including calcium phosphate, E341), especially due to their dissolution in the gastrointestinal tract. Interestingly, this was explicitly stated, despite the reservation that nanoparticles cannot be excluded in phosphates when used as food additive ([78], p. 14). In particular, phosphate salts are considered as safe in food, and there is no danger of acute oral toxicity, genotoxicity, cardiovascular disease, loss of bone mineral density, or carcinogenicity. A daily intake (ADI) of 40 mg P/kg bodyweight per day was considered to be safe. For calcium phosphates, the panel recommended that in the future, the European Commission should request the particle size distribution for these solid powders [78]. Inorganic phosphates are approved in the EU as a food additive in 104 types of food, including baby formulae (see [78] for a full list of cases). Depending on age, people ingest between 350 and 1800 mg phosphorus per day (15 to 90 mg P/kg bodyweight) from food additives (not only calcium phosphate) in their diet [78]. The main sources of phosphate for infants after weaning are infant formulae, cereal-based foods, and baby foods; for adults and elderly persons, the major sources of phosphate are milk, bread, rolls, fine bakery wares, meat, processed cheese, sugars, and syrups [78].

Usually, calcium phosphate is applied as a precipitated inorganic salt, i.e., as monocalcium phosphate, dicalcium phosphate, or hydroxyapatite. Tricalcium phosphate powder is only available by calcination, followed by grinding [3]. It is difficult to find particle size data for food additives, but it is reasonable to assume that usually microparticles and agglomerated nanoparticles are applied. 

After swallowing, all calcium phosphates are dissolved in the stomach under acidic conditions (pH about 1) and converted into the soluble ions Ca^2+^, dihydrogen phosphate H_2_PO_4_^−^, and partially also into phosphoric acid. The solubility at pH 1 neither depends on the particle size nor on the individual calcium phosphate phase. Under the slightly basic conditions in the gut, the ions will precipitate as calcium phosphate again [80]. It must be stressed that there is no difference between calcium ions and phosphate ions from a dissolved calcium phosphate salt and a mixture of these ions that was ingested separately, e.g., by consuming a calcium-rich food such as cheese and a phosphate-rich soft drink together. After dissolution, the ions do not “remember” where they originally came from.

## 7. Calcium Phosphate Nanoparticles in Infant Formulae

The occurrence of small calcium phosphate nanoparticles in infant formulae as reported in 2016 [81,82] is neither surprising, nor does it indicate a risk. Although appearing rather scary in the transmission electron microscopy, small “nano-needles” of calcium phosphate [82]) will rapidly dissolve in an infant’s stomach together with all other calcium phosphate particles. It is also important to note that in the US, the FDA Infant Formula Act requires >60 mg calcium and >30 mg phosphorus per 100 kilocalories of prepared formula [83]. An amount of 30 mg phosphorus (P) corresponds to 92 mg phosphate (PO_4_^3−^). As infant formula has about 5 kcal per g, this corresponds to >3 mg calcium and >4.5 mg phosphate per g, i.e., >7.5 mg calcium phosphate together per g. If 10 g baby formula are dissolved in 90 g water to yield 100 mL milk, the concentration of calcium phosphate is >750 mg per L, which is well above the equilibrium solubility of calcium phosphate [3]. The corresponding requirements for infant formulae in the EU are 50 to 140 mg calcium and 25 to 90 mg phosphorus (equivalent to 77 to 276 mg phosphate) per 100 kcal [84], i.e., the supersaturation may be even higher. It is also noteworthy that the FDA requires a weight ratio of calcium-to-phosphorus between 1.1 and 2.0 (EU: between 1 and 2) [83]. This is a molar ratio of Ca: P between 1:1.3 and 1:2.6, i.e., in the range found in solid calcium phosphates. 

Even if the organic components in the baby formula will prevent the precipitation of calcium phosphate to some degree, it is very likely that calcium phosphate (nano)particles will precipitate during processing and manufacturing of the infant formula and/or after preparation of the infant formula (mixing with water). The product of the ion concentrations in the final baby formula will be well above the solubility product of hydroxyapatite [4]. The (nano)particles will then be swallowed by the infant. As outlined above, this is without any danger, because all calcium phosphate particles present in the infant formula, from nano- to micro-size, will rapidly dissolve in the stomach and be used as a nutritional additive by the body after digestion. This is the underlying reason for the requirements by FDA and EU to require minimum contents of calcium and phosphate in baby formulae. After all, breast milk also contains nanoparticles, as does infant formula.

## 8. Summary and Conclusions

**Figure 2:**
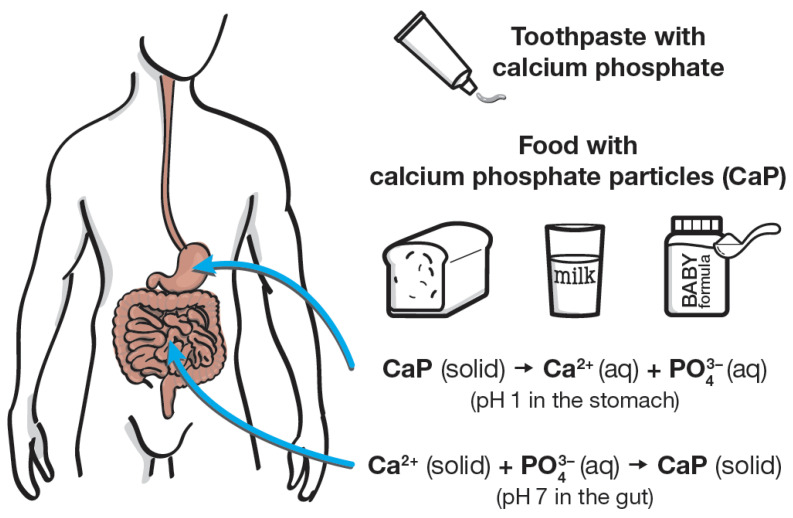
Pathway of calcium phosphate after application as food additive or in toothpaste (this is relevant, e.g., for infants that unintentionally swallow the toothpaste during tooth brushing), together with general aspects on the pathway of calcium phosphate in the human body.

Although calcium phosphate is best known for its applications in biomedicine and in dentistry, it finds many applications in the food industry. The fact that it is a non-toxic, odorless, tasteless, and colorless white powder makes it a well-suited additive, e.g., to enhance the flow properties of flour. It may also be used to neutralize acids in dough, whereupon only the harmless ions calcium and hydrogenphosphate are released. All calcium phosphates, regardless of their particle size (i.e., from nano to micro) are dissolved after ingestion in the stomach. After this dissolution, there is no difference to a swallowed mixture of calcium and phosphate, e.g., from two different foods or drinks. Calcium and phosphate ions are then further processed in the gut. As calcium and phosphate are the constituting ions of human skeletal tissue, they have a positive effect on bone growth and skeletal strength. Consequently, calcium phosphate can be considered as safe for applications in food and in oral care products, regardless of the particle size and the specific calcium phosphate phase. This is underscored by the presence of small calcium phosphate nanoparticles (clusters) in milk (including human breast milk), which is a regular part of our diet, and also their occurrence in blood and in the gut. Figure 2 summarizes the different aspects of calcium phosphate uptake and processing.

In summary, calcium phosphate in particle form has many useful applications in food and dentistry, and the beneficial effects by far exceed any potential risks. Together with the provision of calcium and phosphate ions in the human body, it represents both a useful and a healthy ingredient in food and dental products.

## Data Availability

Not applicable.
